# Safety, Feasibility, and Efficacy of Immersive Virtual Reality Training With a Body Weight Support System for Patients With Extrapyramidal Disorders: A Pilot Case Series Study

**DOI:** 10.7759/cureus.109404

**Published:** 2026-05-21

**Authors:** Kanta Kitabayashi, Naoto Ogura, Naoki Yoshioka, Wataru Kakuda

**Affiliations:** 1 Department of Rehabilitation Medicine, International University of Health and Welfare School of Medicine, Narita, JPN; 2 Department of Rehabilitation Medicine, International University of Health and Welfare Ichikawa Medical Center, Ichikawa, JPN; 3 Department of Radiology, International University of Health and Welfare Narita Hospital, Narita, JPN

**Keywords:** extrapyramidal disorders, japanese rehabilitation, rehabilitation, standing balance, virtual reality training, walking speed

## Abstract

Background

Extrapyramidal disorders, including Parkinson’s disease, multiple system atrophy, and progressive supranuclear palsy, are commonly associated with balance disabilities and an increased risk of falls. Therefore, continuous balance training is important for maintaining quality of life. Recently, rehabilitation using virtual reality (VR) has been introduced; however, most studies have focused on non-immersive VR. In addition, there have been no reports of immersive VR (IVR) training being applied to patients with extrapyramidal disorders. Therefore, we developed a safe, task-oriented standing balance training protocol using IVR combined with a body weight support (BWS) system. The purpose of this study was to evaluate the safety, feasibility, and potential efficacy of IVR training using a BWS system for standing balance and walking speed in patients with extrapyramidal disorders.

Methods

This pilot case series study (a before-after trial) enrolled seven patients with extrapyramidal disorders. All participants were admitted to our rehabilitation ward to receive long-term inpatient rehabilitation. After admission, physical assessments were performed, and participants then underwent 20 minutes of IVR training for standing balance and walking speed combined with conventional physical therapy. This combined protocol was continued for 10 consecutive days (a total of 10 sessions). IVR training was designed to facilitate center-of-gravity shifts and stepping movements in a standing position by requiring participants to reach and touch virtual targets. Visual and auditory feedback were provided, and performance was quantified as a VR score based on the duration of successful target contact. Task difficulty was adjusted according to each participant’s ability. A BWS system was used to ensure safety during training. Berg Balance Scale (BBS), Functional Reaching Test (FRT), and maximum walking speed during the 10-m walking test were assessed as primary outcomes before the first IVR training session and after completing all sessions. Significant differences in the outcomes were determined using the paired Student’s t-test, with a significance level of <0.05. The effect size (ES) was calculated using Cohen’s d.

Results

Among the study participants, five participants completed the 10-day protocol of IVR training, although two participants were discharged before training completion for non-study-related reasons. The total score of the Simulator Sickness Questionnaire was 5.9 ± 5.2, and no serious adverse events were observed during IVR training. No cases of falls were reported. VAS scores for satisfaction, enjoyment, and immersion were high (75.9 ± 19.6, 83.4 ± 17.0, and 81.3 ± 20.4, respectively). The 10-day IVR training promoted significant improvements in the BBS and FRT (p < 0.01). Although no significant improvement was found in maximum walking speed (p = 0.058), a moderate ES was observed (d = 0.69).

Conclusions

Our proposed IVR training using a BWS system could safely and feasibly improve standing balance and may contribute to improvements in walking speed among patients with extrapyramidal disorders. Further studies with larger sample sizes are needed to confirm the efficacy of this training.

## Introduction

Extrapyramidal disorders, including Parkinson’s disease (PD), multiple system atrophy (MSA), and progressive supranuclear palsy (PSP), are progressive conditions characterized by the gradual loss of neuronal function [1-3]. Although these diseases present with a variety of motor and non-motor symptoms, a common feature is balance and walking disabilities, which are associated with an increased risk of falls [1-3]. The increased risk of falls often heightens patients’ fear of falling, which in turn can lead to limited physical activity, social withdrawal, and a decline in quality of life (QOL) [4]. In addition, a higher prevalence of depression and anxiety symptoms has been reported in these conditions [5]. Therefore, to maintain physical function and QOL in patients with extrapyramidal disorders, it is necessary to explore feasible and acceptable training methods.

Recently, novel techniques such as virtual reality (VR) training have emerged as therapeutic tools for patients with stroke and PD, not only in Japan but also in the world [6-9]. However, most previous studies have focused on the effectiveness of training using non-immersive VR systems, such as computer displays, console gaming systems, and commercially available games, primarily due to safety considerations and ease of implementation [7]. A recent systematic review and meta-analysis in patients with chronic stroke reported that none of the 43 included studies employed immersive VR (IVR) using head-mounted displays (HMDs) for dynamic balance training [10]. These findings indicate that research on IVR-based interventions targeting balance and walking remains limited, and their effectiveness has not yet been fully established. Furthermore, there are no reports investigating the use of IVR training in patients with PSP or MSA. Therefore, the development of IVR-based training methods that enable patients with extrapyramidal disorders to safely train standing balance and walking speed is warranted. For this reason, we originally developed a novel protocol for standing balance and walking speed training using an IVR system in combination with a body weight support (BWS) system. We expect that the combined use of a BWS system could ensure the safety of the training and reduce patients’ fear of falling, leading to higher efficacy of the training.

The purpose of this pilot case series study was to explore the safety, feasibility, and potential efficacy of our proposed IVR training with a BWS system for standing balance and walking speed in patients with extrapyramidal disorders. We hypothesized that our proposed IVR training would be safe and feasible and may improve balance, walking speed, and fear of falling in patients with extrapyramidal disorders.

## Materials and methods

Participants

This study enrolled a total of seven patients with extrapyramidal disorders. All participants were admitted to our rehabilitation ward to receive long-term inpatient rehabilitation between April 2024 and September 2025. The inclusion criteria for this study were as follows: (1) clinical diagnosis of extrapyramidal disorders presenting with mild-to-moderate balance disability upon admission to our ward; (2) age between 20 and 90 years upon admission; (3) ability to maintain sitting and standing position for a couple of minutes; (4) no severe cognitive impairment; (5) no severe visual impairment; (6) no history of seizures; (7) no severe freezing of gait; (8) no orthopedic or medical diseases that could significantly limit physical activity; (9) no severe orthostatic hypotension due to autonomic neuropathy; (10) no severe dizziness at rest; (11) ability to understand the purpose and protocol of this study; and (12) ability to provide written informed consent for study inclusion.

The protocol of our VR training and some evaluations conducted in this study were approved by the Ethics Committee of the International University of Health and Welfare (approval number: 23-Im-017-2) and registered with the University Hospital Medical Information Network (UMIN) Center (UMIN-CTR ID: R000060821, UMIN000053291). This study was conducted in compliance with the Declaration of Helsinki, and written informed consent was obtained from all participants before study inclusion.

Intervention

This was a pilot case series study (a before-after trial). This study investigated the effects of IVR training in combination with conventional physical therapy. First, after admission to our ward, a physical assessment was performed. After the initial assessment, each IVR training session lasted 20 minutes and was provided for the recovery of standing balance and walking ability, along with conventional physical therapy (muscle strength training, balance training, walking training, stair-climbing training, etc.). The content and intensity of conventional physical therapy were adjusted according to each participant’s physical function and clinical condition. IVR training continued for 10 consecutive days (10 sessions in total), with one session conducted per day. One session consisted of two sets, each with a maximum of five minutes, depending on the participant’s ability. During training, an approximately three-minute break was scheduled between sets, during which blood pressure, heart rate, and other vital signs were assessed as needed.

Throughout the hospitalization period, all participants were continuously monitored by the attending physicians and physical therapists through clinical and neurological examinations, with special attention paid to the development of adverse events associated with IVR training, such as dizziness, nausea, and seizures. Because all participants were admitted primarily for respite care or inpatient rehabilitation, medication regimens were generally maintained throughout the admission period and were not systematically adjusted for this study. Although minor medication adjustments were made for some participants based on clinical needs, no major changes to antiparkinsonian treatment were implemented during the intervention period. In addition, to minimize the influence of daily motor fluctuations, IVR training sessions and outcome measures were conducted at approximately the same time of day for each participant.

**Figure 1:**
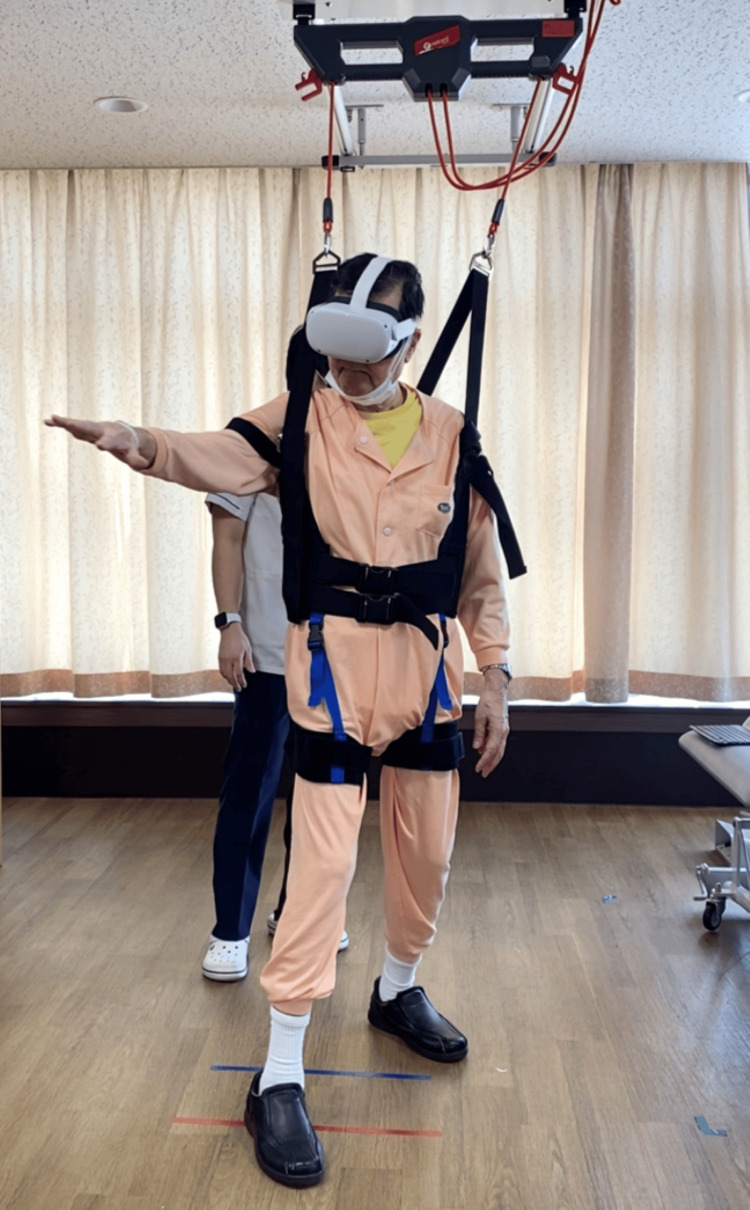
IVR training with a BWS system Participants were fitted with a BWS system to prevent falls during training and were carefully monitored by a trained physical therapist. BWS, body weight support; IVR, immersive virtual reality

This study used Meta Quest 2 (Meta Platforms, Menlo Park, CA, USA), with images projected onto an iPad (Apple, Cupertino, CA, USA), which allowed the therapist to simultaneously monitor the virtual space during training. This IVR training was conducted without holding the controller. Importantly, all participants were equipped with a BWS system to ensure safe performance of high-difficulty balance tasks in the standing position and prevention of falls during training, as shown in Figure 1. The BWS system used in this study was a ceiling-mounted device comprising an adjustable rope and a trunk harness. Before each session, a physical therapist fitted the participants with the harness, which was securely fastened around the participant’s trunk and hips. The rope length was adjusted according to each participant’s height, allowing for natural posture and movement. The BWS system was used only as a safety measure to prevent falls, and no fixed body weight unloading was applied during training.

**Figure 2:**
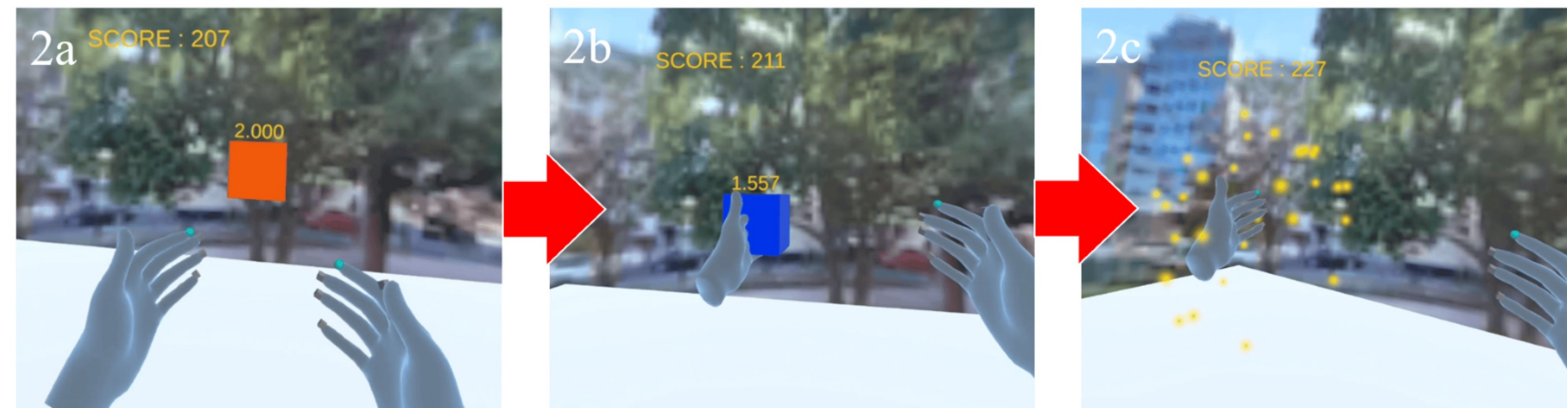
Typical image of the virtual environment during training A projection of the participant’s hands and orange targets on the virtual image (a). When the participant successfully reaches and touches the target, the color of the target changes (b). Finally, if the target is touched for two seconds, a small yellow explosion appears, followed by a distinct sound produced by the Meta Quest 2 speaker (c).

During the IVR training, both the participant’s hands and orange box targets were presented on the IVR display, as shown in Figure 2a. Thereafter, participants were instructed to touch the static and dynamic box-shaped targets that appeared vertically and horizontally with their left and right hands. During training, participants were verbally encouraged by the physical therapist to actively reach toward the targets and perform stepping movements when the targets were beyond arm’s reach. This IVR training method was designed to facilitate center-of-gravity shift and stepping movements in the standing position by requiring participants to touch virtual targets. The targets appeared at random positions and timings within the participant’s reachable space to encourage multidirectional weight shifting and upper-limb reaching movements during standing. In addition, the targets were designed to always pass in front of the participant to reduce the time required for visual searching and increase the frequency of weight-shifting and stepping movements during training. No manual assistance for upper-limb movement or balance control was routinely provided during the task. Physical assistance was provided only when a participant was judged to be at imminent risk of falling despite the use of the BWS system.

As feedback to the participant, the color of the target changed when successfully touched, as shown in Figure 2b, 2c, followed by a distinct sound. Moreover, the time that participants were able to touch in one set was displayed as the VR score. The IVR score adds one point for every 0.1 seconds during which the target is successfully touched by the participant’s hand.

For IVR training, two difficulty levels, “easy mode” and “normal mode,” were prepared. At first, easy mode was applied for all participants. A higher score indicated a longer duration of successfully touching the target within a single set. The task progressively became more difficult as the VR score improved, specifically when a participant scored at least 1000 points for one set on two consecutive days. As the difficulty is increased, the reaching distance is extended, and the targets appeared on the image more frequently and moved faster. The VR application for this study was uniquely created on Unity 2021.3.1f1 (Unity Technologies, San Francisco, CA, USA) with study collaborators.

Primary outcomes

To evaluate physical function, three objective assessment scales, including the Berg Balance Scale (BBS), Functional Reaching Test (FRT), and 10-m walk test, were applied. These scales were evaluated by the same physical therapist who conducted the IVR training sessions before the first IVR training session and after the 10 sessions; therefore, assessor blinding was not implemented in this study. The BBS, which comprehensively assesses static and dynamic balance ability, has a maximum total score of 56 [11]. The FRT measures the distance (cm) of maximal forward reach in the standing position [12]. Participants must stretch their arms forward as far as possible while maintaining the base of support. Achieving a longer distance on this test indicates better balance ability. The maximum walking speed was evaluated using a 10-m walk test [13]. Participants were evaluated before and after IVR training under similar measurement conditions (if needed, walking aids were applied) to increase the reliability of the evaluation results.

Secondary outcomes

Baseline clinical characteristics were assessed using the Hoehn and Yahr (H&Y) stage [14], Functional Ambulation Categories [15], and Barthel Index [16]. We also evaluated three self-assessment scales: the Simulator Sickness Questionnaire (SSQ), the Visual Analogue Scale (VAS), and the Fall Efficacy Scale (FES). The SSQ was used to evaluate adverse effects associated with the proposed training based on 16 symptoms (i.e., headache, nausea, and eyestrain) on a 4-point Likert scale (none = 0, slight = 1, moderate = 2, or severe = 3) [17,18]. The SSQ provides specific sub-scores for nausea, oculomotor disturbance, and disorientation, with their sum yielding the total score. SSQ scores were calculated according to the original methodology outlined by Kennedy et al. [17]. Accordingly, a higher total SSQ score indicates more severe adverse effects. The VAS was applied to evaluate participants’ satisfaction, enjoyment, and immersion in our proposed training. The VAS scores were determined using a 10-cm horizontal line with a “score of 0” (i.e., “no satisfaction, enjoyment, and immersion”) written on the left end, whereas a “score of 100” (i.e., “highest satisfaction, enjoyment, and immersion”) was written on the other end [19,20]. The SSQ and VAS scores were evaluated after completing all 10 IVR training sessions. Furthermore, the FES was applied to evaluate fear of falling by determining participants’ confidence in performing a 10-item activity without falling. The scores were graded from 10 to 40, with lower scores indicating greater fear of falling [21]. The FES was evaluated before the first IVR training session and after completing all 10 sessions.

Statistical analysis

Data were expressed as mean ± SD. Disease severity was categorized into three levels (mild, moderate, and severe) based on clinical presentation and functional status. Cognitive function was categorized into four levels (normal, mildly impaired, moderately impaired, and severely impaired) based on overall clinical assessment. Significant differences in the BBS score, FRT score, maximum walking speed, and FES score before and after the IVR training protocol were determined using the paired Student’s t-test. A p-value of <0.05 indicated statistical significance. The effect size (ES) was calculated using Cohen’s d. All statistical analyses were performed using IBM SPSS Statistics for Windows, version 23.0 (released 2015; IBM Corp., Armonk, NY, USA).

## Results

Table 1 summarizes the qualitative clinical characteristics of the participants, including diagnosis and disease severity. Table 2 presents the quantitative clinical characteristics. The mean age at admission was 72.7 ± 8.0 years, whereas the duration between diagnosis and admission was 4.8 ± 5.0 years. The mean score of the Barthel Index, which served as an initial clinical evaluation before IVR training (before the first session), was 66.4 ± 14.4 points. In terms of disease severity, six participants were classified as moderate (6/7, 85.7%) and one as mild (1/7, 14.3%), whereas only one participant (1/7, 14.3%) showed cognitive impairment.

**Table 1:** Qualitative clinical characteristics of the participants Data are presented as individual values for each participant. Disease severity (mild, moderate, and severe) and cognitive function (normal, mildly impaired, moderately impaired, and severely impaired) were classified based on clinical assessment, including functional status and symptom severity. H&Y, Hoehn and Yahr; IVR, immersive virtual reality; MSA-C, multiple system atrophy, cerebellar type; PD, Parkinson’s disease; PSP, progressive supranuclear palsy

Patient no.	1	2	3	4	5	6	7
Gender (M/F)	M	F	M	F	F	M	M
Diagnosis	PSP	PD	MSA-C	MSA-C	PD	PD	PD
Disease severity	Mild	Moderate	Moderate	Moderate	Moderate	Moderate	Moderate
H&Y stage (1-5)	-	3	4	3	3	4	3
Functional ambulation categories before IVR training	3	3	2	3	4	2	4
Walking aids	No need	No need	Walker	Walker	Cane	Walker	No need
Cognitive function	Normal	Moderately impaired	Normal	Normal	Normal	Normal	Normal

**Table 2:** Quantitative clinical characteristics of the participants Data are presented as individual values for each participant. IVR, immersive virtual reality

Patient no.	1	2	3	4	5	6	7
Age at admission (years)	73	84	58	70	78	72	74
Disease duration (years)	1	Not available	4	1	7	2	14
Barthel Index before IVR training (points)	70	65	45	75	85	50	75
Time for one set of IVR training (minutes)	5	3	3	5	5	5	5

**Figure 3:**
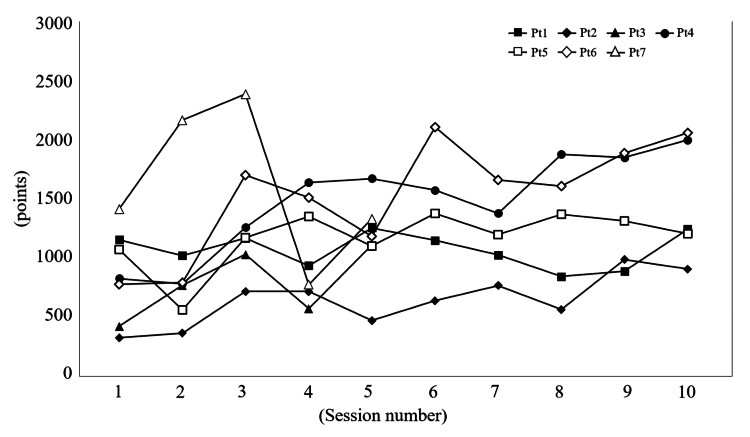
Changes in VR scores per session Data are presented as individual values. To adjust the difficulty level, sessions 4 and 5 for patient no. 7 were conducted in “normal mode.” VR, virtual reality

Among the seven participants, two (2/7, 28.6%; patients nos. 3 and 7) were discharged after five sessions for non-study-related reasons, whereas the other five completed the scheduled 10-day training. For two participants (2/7, 28.6%; patients nos. 2 and 3), the duration of one set was set to three minutes (one session: six minutes) due to high levels of fatigue, whereas the remaining five (5/7, 71.4%) participants completed the five-minute (one session: 10 minutes) protocol. The VR score per session for each participant is shown in Figure 3. All participants demonstrated a gradual increase in scores over time, indicating a longer duration of successful reaching and target contact without loss of balance. Only one participant (1/7, 14.3%; patient no. 7) exceeded 1000 points per set in easy mode and subsequently continued training in normal mode. 

Comparison of primary outcome measures

**Figure 4:**
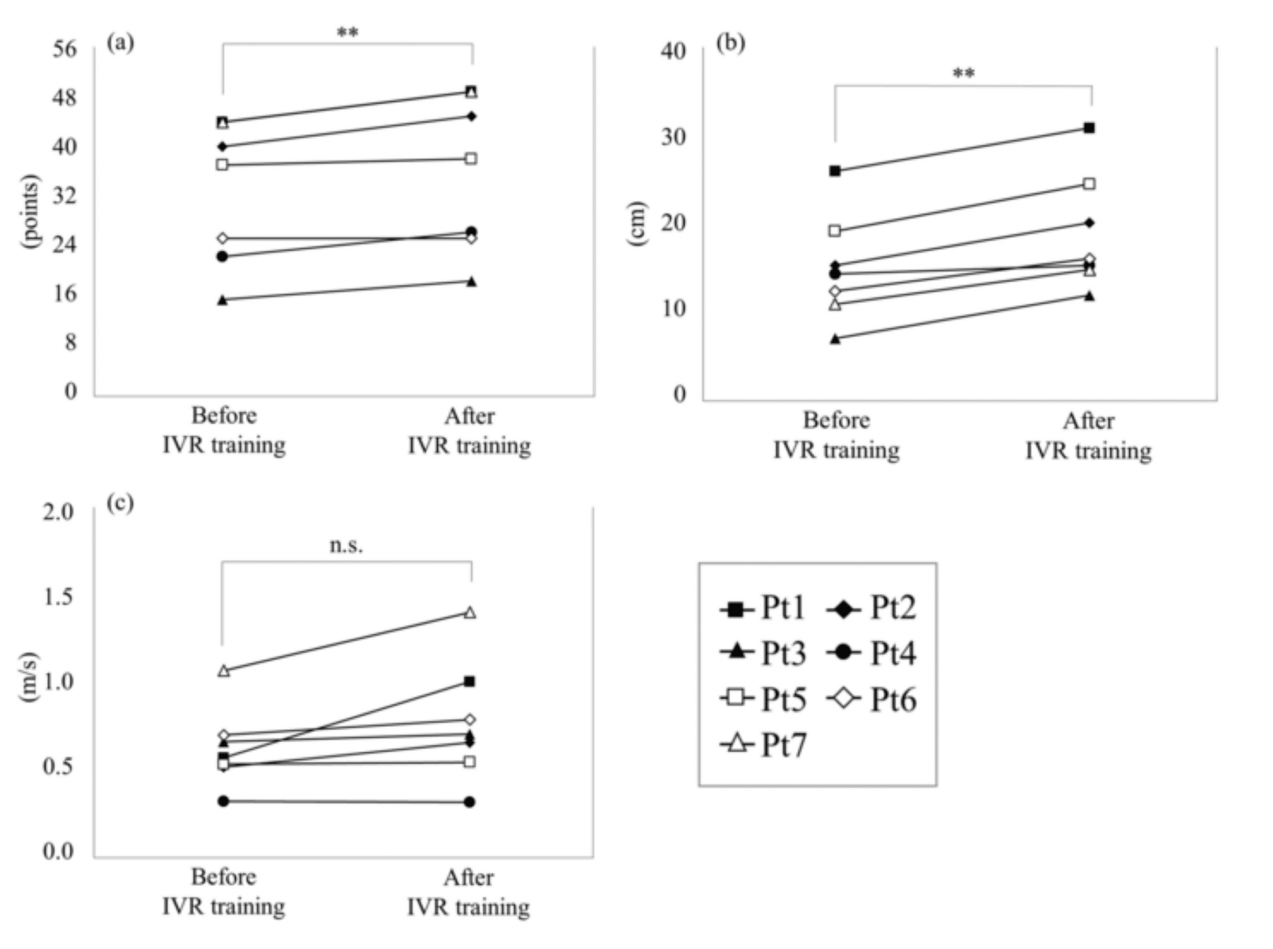
Changes in physical function with the proposed IVR training protocol Total BBS score (a), distance in FRT (b), and maximum walking speed (c). Data are presented as individual values. A paired t-test was used to compare values before and after IVR training. Statistical significance was set at p < 0.05. ** indicates a significant difference between conditions at p < 0.01; n.s., not significant. BBS, Berg Balance Scale; FRT, Functional Reaching Test; IVR, immersive virtual reality

Figure 4 shows the changes in the three primary outcome measures with training. Accordingly, the changes in BBS and FRT parameters suggested potential improvements associated with the proposed IVR training. The BBS score increased significantly from 32.4 ± 11.6 points to 35.7 ± 12.7 points after training (mean difference: 0.78; 95% CI: -5.2 to -1.4; t = -4.2; ES: 0.87; p < 0.01) (Figure 4a). Moreover, the reach distance during the FRT significantly increased from 14.7 ± 6.3 to 18.9 ± 6.8 cm (mean difference: 0.57; 95% CI: -5.6 to -2.8; t = -7.2; ES: 0.95; p < 0.01) (Figure 4b). However, walking speed did not significantly increase from 0.6 ± 0.2 to 0.8 ± 0.4 m/s (mean difference: 0.06; 95% CI: -0.3 to -0.007; t = -2.3; p = 0.058) (Figure 4c). Nevertheless, it did present a medium-sized effect (d = 0.69).

Comparison of secondary outcome measures

Table 3 shows the SSQ score after IVR training regarding adverse effects. The average score for the total SSQ was 5.9 ± 5.2. Furthermore, the mean SSQ sub-scores for nausea, oculomotor, and disorientation were 1.4 ± 3.6, 8.7 ± 6.8, and 6.0 ± 11.0, respectively. The most common adverse effect was “fatigue,” which was reported by four participants (4/7, 57.1%). Other adverse effects experienced by the participants included “eyestrain” (2/7, 28.6%), “fullness of head” (1/7, 14.3%), “difficulty concentrating” (1/7, 14.3%), and “dizzy” (1/7, 14.3%), all of which were mild and transient and did not require any medical care. Moreover, no falls were reported during IVR training in this study. VAS scores for satisfaction, enjoyment, and immersion for our protocol were 75.9 ± 19.6, 83.4 ± 17.0, and 81.3 ± 20.4, respectively, which suggested that our training promoted at least favorable levels of satisfaction, enjoyment, and immersion. The mean FES score significantly increased with our training (from 26.3 ± 7.2 to 28.1 ± 6.9 points; mean difference: 0.6; 95% CI: -3.3 to -0.4; t = -3.1; ES: 0.79; p < 0.05), suggesting a possible reduction in fear of falling following the intervention.

**Table 3:** SSQ scores for participants Each value in parentheses indicates the score range. Data are presented as individual values for each participant. SSQ, Simulator Sickness Questionnaire

Patient no.	1	2	3	4	5	6	7
Total score (0-179.52)	7.5	7.5	15	0	7.5	3.7	0
Nausea (0-200.34)	0	0	0	0	9.5	0	0
Oculomotor (0-159.18)	15.2	15.2	15.2	0	7.6	7.6	0
Disorientation (0-292.32)	0	0	27.8	0	13.9	0	0

## Discussion

The current study suggests that our proposed IVR training with a BWS system may be safe, feasible, and potentially beneficial for standing balance in patients with extrapyramidal disorders. To the best of our knowledge, this study is the first to report that IVR training with a BWS system was significantly associated with improvements in standing balance in patients with extrapyramidal disorders, including MSA and PSP.

The total SSQ score in this study was 5.9, indicating that the training protocol was completed safely with no serious adverse effects. Saredakis et al. reported that SSQ scores associated with HMDs range from 14.3 to 35.3 [22], suggesting that the incidence of adverse effects in our study was relatively low. Such effects are generally attributed to mismatches between visual and vestibular information [23]. To mitigate these risks, the target objects were designed to pass in front of the participants, minimizing head rotation. In addition, the short training duration (a maximum of five minutes per session, up to 10 minutes per day) may have contributed to the low incidence of adverse effects, as prolonged IVR exposure is known to increase these adverse effects [24]. These factors likely allowed participants to complete the protocol safely.

Satisfaction, enjoyment, and immersion showed high VAS scores after the IVR training protocol. Levin demonstrated that providing immediate feedback in response to participants’ movements is important for maintaining motivation [25]. In our IVR training, visual and auditory feedback were provided upon successful target contact, which may have promoted participation. Therefore, this IVR training may represent a feasible long-term intervention for patients with neurodegenerative diseases. Notably, one participant with moderate cognitive decline (patient no. 2) was able to complete the protocol, suggesting its versatility and potential advantage over non-immersive VR, as it allows intuitive training without complex operations.

The current study showed a significant improvement in standing balance, as evidenced by the BBS and FRT scores, with a large ES (d = 0.87 and 0.95). Furthermore, although no significant difference was observed in maximum walking speed, a moderate ES was noted. Patients with PSP, MSA, and PD generally exhibited balance impairments due to dysfunction in neural regions such as the substantia nigra, cerebellum, and basal ganglia [1,26,27]. These lesions are known to increase the risk of falls by impairing anticipatory postural adjustments (APAs) [28,29], which can be improved through repeated training under various conditions and movements. Our proposed IVR training may have provided task-specific standing balance training targeting APAs by having participants perform reaching movements toward targets moving in various directions. Repeated reaching movements in response to targets appearing at different locations may have contributed to the observed improvements in balance. In addition, improvements in balance were associated with a moderate ES for walking speed. Nevertheless, the inclusion of participants who used walking aids might have limited the ability to detect a statistically significant difference. Most importantly, the combination of IVR with a BWS system ensured training safety, thereby enabling patients to participate in balance training in a reassuring environment.

In this study, as shown in Figure 3, the VR scores gradually improved across daily sessions. In addition, this IVR training protocol was associated with a significant improvement in FES scores. Feng et al. suggested that one advantage of IVR is the provision of abundant feedback and quantitative rewards during task performance [30]. Such features may help patients with neurodegenerative diseases recognize their performance and progress, thereby promoting active participation in rehabilitation. Furthermore, improvements in VR scores might enhance patients’ confidence in their abilities, which may translate into greater confidence in activities of daily living. Therefore, IVR training might serve as a useful tool for sustaining physical activity by improving exercise adherence and encouraging continued participation in rehabilitation.

This study has certain limitations worth noting. First, this was a pilot case series study with only a small number of patients and no control group. Comparative studies, such as randomized controlled studies that include a large number of patients, are needed to confirm the efficacy of our proposed IVR training in patients with extrapyramidal disorders. Second, the long-term effects of our proposed IVR training were not evaluated. Accordingly, future studies should examine whether the beneficial effects of our training could be maintained after cessation of the 10-day IVR training. In addition, it will be important to investigate potential adverse events and the impact on standing balance when IVR training is conducted for periods longer than the 10-day protocol. Third, we did not apply any neuroimaging investigations, such as functional magnetic resonance imaging and near-infrared spectroscopy, which could be important for clarifying the underlying mechanisms by which our proposed IVR training promoted functional recovery.

## Conclusions

This pilot case series study suggests that our proposed IVR training with a BWS system can be performed safely by patients with extrapyramidal disorders. Furthermore, this training may improve standing balance and walking speed in these patients. In addition, the high level of satisfaction suggests that this training may be feasible for continued use and may serve as a useful tool. Further large-scale studies are needed to confirm the therapeutic benefits of this IVR training and to determine the optimal training duration and frequency for patients with extrapyramidal disorders.
